# 11.G. Round table: Resilient health systems: harnessing health information to improve population health

**DOI:** 10.1093/eurpub/ckac129.715

**Published:** 2022-10-25

**Authors:** Organised by: EUPHA-PHMR, EUPHA-HSR, EUPHA-ECO, Sciensano (Belgium), PHIRI, Chair persons: Petronille Bogaert (EUPHA-PHMR), Johan Hansen (EUPHA-HSR)

## Abstract

Health systems are built to improve the health of the population. When the COVID-19 crisis hit Europe, the sustained performance of these health systems was challenged. The resilience of these health systems, defined as the ability to absorb, adapt, and transform to cope with shocks (Observatory, 2021), was found to be different in the many European countries, leaving some important lessons to be learned and best practices to be showcased to help countries assess their own response to the COVID-19 pandemic and support efforts to strengthen health systems in Europe. A common denominator is health information; the data and information that is needed to monitor the health of patients as well as the general population. Especially in times of crisis, the availability and trustworthiness of these data is of utmost importance. The COVID-19 pandemic showed that within a substantial number of European countries, health information systems were not always equipped to accommodate the data and information flows that were needed in order for researchers to provide the best available evidence to underpin health policy decisions. Ad-hoc surveillance and monitoring systems were set up (under emergency legislation) and clear governance of health information was lacking. In addition, sharing data and information across European borders and ensuring comparability of data and indicators proved to be difficult in a timely manner during the COVID-19 crisis. This resulted in a European landscape with different national and federal health policies, based on sometimes poor scientific findings. However, when the crisis progressed, numerous national and international initiatives were set up aiming to harmonize the available health information and as of now, many of these initiatives are forming a solid foundation of the health systems, rendering their performance sustainable for the future. In this workshop, which is organized as a round table discussion, we will discuss the resilience of health information systems in European countries, with regards to lessons learned from the COVID-19 crisis, barriers to sharing health information within and across borders, best practices and future perspectives. The topic will be highlighted from multiple perspectives, bringing together experts from different backgrounds, including the European level (European Commission and European Observatory on Health Systems and Policies), the country level, and the perspective of European projects. The audience will be able to provide their view on the different topics through an interactive voting poll during the session. Throughout the session, the exchange of knowledge, experiences and opinions with the audience will be facilitated by the chairs.

**Key messages:**

• Population health information plays a key role in times of crisis, with trustworthy information flows facilitating evidence-informed policies and decision-making.

• Sharing and harmonising health information is key to building resilient healthcare systems that are prepared for the future.

**Speakers/Panellists:**

**Ebba Barany**

European Commission, Luxembourg, Luxembourg

**Cianrán Nicholl**

European Commission, JRC, Ispra, Italy

**Kenneth Grech**

Public Health Medicine, Mater Dei Hospital, L-Imsida, Malta

**Miriam Saso**

Sciensano, Brussels, Belgium

**Anna Sagan**

European Observatory on Health Systems and Policies, London, UK

